# Prognostic value of serum amyloid A in COVID-19: A meta-analysis

**DOI:** 10.1097/MD.0000000000028880

**Published:** 2022-02-18

**Authors:** Yongkai Li, He Xiaojing, Li Zhuanyun, Dandan Li, Jianzhong Yang

**Affiliations:** a Emergency Trauma Center, the First Affiliated Hospital of Xinjiang Medical University, Urumqi, China; b Seven Section of Department of Gynecology, The Second Hospital of Hebei Medical University, Shijiazhuang, Hebei, China.

**Keywords:** coronavirus disease 2019, meta-analysis, serum amyloid A

## Abstract

**Background:**

There is still a lack of large-scale clinical studies and evidence-based evidence to prove the relationship between serum amyloid A (SAA) and the severity and prognosis of patients with new coronavirus pneumonia (COVID-19).

**Methods:**

We searched PubMed, Cochrane Library, Excerpta Medica Database, and Web of Science for original articles from December 1, 2019 to December 19, 2020. Search criteria include free text search, explosive MESH/EMTREE terms, and all synonyms for SAA and COVID-19. There are no language restrictions on the searched documents. Statistical methods were performed using Stata 14.0 software, and RevMan 5.4 software provided by the Cochrane Collaboration for meta-analysis. The 10 included studies in the literature were classified according to the severity of the novel coronavirus treatment guidelines, with mild/moderate categorized as nonsevere and severe/critical as severe, and the data were meta-analyzed using multiple subgroup standard deviations combined. Severe and nonsevere were finally divided into 2 groups, and the combined data were meta-analyzed according to the standardized mean difference.

**Results:**

The results of the meta-analysis given by random effects showed that SAA levels were significantly higher in severe vs nonsevere (standardized mean difference 1.20 [95% confidence interval 0.91–1.48]), which was statistically significant (*P* < .001). The 3 literatures studied (random effect size 0.11 [95% confidence interval 0.05–0.19]; I^2^ = 56.68%) and were statistically significant, z = 5.46 *P* < .01, suggesting that the risk of death occurs at higher levels with increasing SAA values, with the risk of death in the severe group being 11% higher than in the nonsevere group.

**Conclusion:**

SAA can be considered as a biomarker for predicting the severity and prognosis of COVID-19. SAA can be used for early warning of the poor prognosis of COVID-19 and for monitoring the recovery process, which has important clinical value.

## 1 Introduction

Coronavirus disease 2019 (COVID-19) is a novel respiratory and systemic disease caused by severe acute respiratory syndrome coronavirus type 2 (SARS-CoV-2) that is currently in epidemic proportions. Globally, as of December 27, 2020, over 4 million new COVID-19 cases and 72,000 new deaths were reported. This brings the cumulative numbers to over 79 million reported cases and over 1.7 million deaths globally since the start of the pandemic.^[1]^ The main manifestations include fever, dry cough, and fatigue. Some patients have diminished or lost sense of smell and taste as the first symptoms. A few patients have symptoms such as nasal congestion, runny nose, sore throat, conjunctivitis, myalgia, and diarrhea. Severe patients often develop dyspnea and/or hypoxemia 1 week after the onset of onset. In severe cases, they can quickly progress to acute respiratory distress syndrome, septic shock, difficult to correct metabolic acidosis and coagulation dysfunction, organ failure etc.^[2]^ As an important inspection method, medical laboratories provide important contributions to the clinical decision-making of this disease and many other infectious diseases.^[3]^ Serum amyloid A (SAA) is an index that has been widely used clinically in recent years and can reflect the infection of the body. Elevated SAA is mainly seen in viral infections, cardiovascular diseases, transplant rejection, and so on. SAA is an acute-phase reactive protein, which can rapidly increase about 1000-fold in the acute phase after the body is infected, and then rapidly decrease to normal level after the pathogen is cleared, and is a sensitive indicator of the body's infection and inflammation recovery.^[^4^,^^5^^]^ SAA levels have a certain correlation with the severity of COVID-19 patients, and the specific positive correlation is helpful for the diagnosis of COVID-19 severity and prognostic evaluation can be used as a risk factor for predicting the fatality rate of COVID-19. Many observational studies have been performed on the relevance of elevated SAA and COVID-19 severity and prognostic value. However, the lack of statistical support and conflicting results did not result in any firm conclusions being drawn, and there was large heterogeneity in the analysis of the results.^[6]^ Thus the value of SAA elevation in this context is still debated. The purpose of this meta-analysis is to draw more reliable conclusions about the relationship between changes in SAA levels and the severity and prognosis of COVID-19 patients.^[7]^

## 2 Methods

### 2.1 Protocol and registration

The purpose of this meta-analysis is to understand the relationship between SAA levels and the severity and prognosis of COVID-19 patients and draw more reliable conclusions. We searched PubMed, Cochrane Library, Excerpta Medica Database, and Web of Science for original articles from December 1, 2019 to December 19, 2020. Search criteria include free text search, exploding MESH/EMTREE terms, and all synonyms for SAA and COVID-19. There are no language restrictions on the searched documents. After exclusion criteria, 10 studies were included in the final analysis, The 10 included studies in the literature were classified according to the severity of the novel coronavirus diagnosis and treatment guidelines, categorizing mild/moderate as nonsevere and severe/critical as severe, and COVID-19 severity was categorized further for the analysis. This meta-analysis was conducted in accordance to the preferred reporting items for systematic reviews and meta-analyses guidelines.^[8]^ An a priori protocol was designed and registered (international prospective register of systemic reviews identification: CRD42020227284).

#### 2.1.1 Inclusion criteria

##### 2.1.1.1 The article type was a retrospective cohort study or a clinical observational study

##### 2.1.1.2 Novel coronavirus nucleic acids were detected in nasopharyngeal swabs, sputum, lower respiratory secretions, blood, stool, and other specimens using reverse transcription-polymerase chain reaction and/or next-generation sequencing methods. Patients were diagnosed with COVID-19 and had positive results of SARS-CoV-2 Ribonucleic Acid

##### 2.1.1.3 The study results show the relationship between SAA levels and patient survival prognosis

##### 2.1.1.4 Report death as the primary endpoint of the study

#### 2.1.2 Exclusion criteria

##### 2.1.2.1 Animal experiments, conference abstracts, case reports, reviews, and other nonclinical research literature

##### 2.1.2.2 Duplicate literature, poor quality research, research literature on the same cohort

##### 2.1.2.3 Literature with incomplete data or inability to obtain valid data

##### 2.1.2.4 No literature on survival prognostic data

### 2.2 Search strategy

For PubMed, the terms were: ((“COVID-19”[Mesh]) OR (((((((((((((((((((((((((((((((((((COVID 19[Title/Abstract]) OR (COVID-19 Virus Disease[Title/Abstract])) OR (COVID 19 Virus Disease[Title/Abstract])) OR (COVID-19 Virus Diseases[Title/Abstract])) OR (Disease, COVID-19 Virus[Title/Abstract])) OR (Virus Disease, COVID-19[Title/Abstract])) OR (COVID-19 Virus Infection[Title/Abstract])) OR (COVID 19 Virus Infection[Title/Abstract])) OR (COVID-19 Virus Infections[Title/Abstract])) OR (Infection, COVID-19 Virus[Title/Abstract])) OR (Virus Infection, COVID-19[Title/Abstract])) OR (2019-nCoV Infection[Title/Abstract])) OR (2019 nCoV Infection[Title/Abstract])) OR (2019-nCoV Infections[Title/Abstract])) OR (Infection, 2019-nCoV[Title/Abstract])) OR (Coronavirus Disease-19[Title/Abstract])) OR (Coronavirus Disease 19[Title/Abstract])) OR (2019 Novel Coronavirus Disease[Title/Abstract])) OR (2019 Novel Coronavirus Infection[Title/Abstract])) OR (2019-nCoV Disease[Title/Abstract])) OR (2019 nCoV Disease[Title/Abstract])) OR (2019-nCoV Diseases[Title/Abstract])) OR (Disease, 2019-nCoV[Title/Abstract])) OR (COVID19[Title/Abstract])) OR (Coronavirus Disease 2019[Title/Abstract])) OR (Disease 2019, Coronavirus[Title/Abstract])) OR (SARS Coronavirus 2 Infection[Title/Abstract])) OR (SARS-CoV-2 Infection[Title/Abstract])) OR (Infection, SARS-CoV-2[Title/Abstract])) OR (SARS CoV 2 Infection[Title/Abstract])) OR (SARS-CoV-2 Infections[Title/Abstract])) OR (COVID-19 Pandemic[Title/Abstract])) OR (COVID 19 Pandemic[Title/Abstract])) OR (COVID-19 Pandemics[Title/Abstract])) OR (Pandemic, COVID-19[Title/Abstract]))) AND ((“Serum Amyloid A Protein”[Mesh]) OR (((((((((((((Amyloid A Protein-Related Serum Component[Title/Abstract]) OR (Amyloid A Protein Related Serum Component[Title/Abstract])) OR (Amyloid Protein AA Precursor[Title/Abstract])) OR (Serum Amyloid Protein A[Title/Abstract])) OR (Amyloid Serum Protein SAA[Title/Abstract])) OR (Amyloid-Related Serum Protein (SAA))) OR (Serum A Related Protein[Title/Abstract])) OR (Serum Amyloid A[Title/Abstract])) OR (Amyloid A Precursor[Title/Abstract])) OR (Amyloid Protein SAA[Title/Abstract])) OR (Amyloid A Protein[Title/Abstract])) OR (Amyloid Fibril Protein AA[Title/Abstract])) OR (Amyloid Protein AA[Title/Abstract]))).

### 2.3 Study selection and data extraction

Two researchers (Li Yongkai and He Xiaojing) independently extracted data for each document that met the inclusion criteria. If there is a difference, it will be resolved through discussion or by a third researcher. Other researchers performed the data collation work.

The extracted data include: first author, research method, sample size, severity, gender, age, SAA (mg/L), and quality assessment. A table was created using Microsoft Excel 2016 (spreadsheet software, Microsoft Corporation) to extract and record the data from the literature.

### 2.4 Statistical analysis

Statistical methods were performed using Stata 14.0 software (StataCorp LLC 4905 Lakeway Drive College Station, USA), and Review Manager analysis software (RevMan 5.4; Cochrane Collaboration, Oxford, UK) for meta-analysis. If the original research data is the median and interquartile range or the range represents continuous variables, The method proposed by Liu et al^[9]^ was used to calculate (x ± s) and estimate standardized mean difference (SMD) or weighted mean difference. Using the meta-analysis method to merge the data by combining the standard deviations of different subgroups.^[10]^ The Q test (test level α = 0.1) combined with I^2^ was used to determine the magnitude of statistical heterogeneity between the literature. Taking *P* ≥ .10 and I^2^ < 50% were considered as no statistical heterogeneity. The fixed-effects model was used for meta-analysis; taking *P* < .10 or I^2^ ≥ 50% is statistically heterogeneous, analyze the source of heterogeneity, after excluding the influence of obvious clinical heterogeneity, using the random-effects model for meta-analysis; when there is obvious clinical heterogeneity, use subgroup analysis or sensitivity analysis or only descriptive analysis.

### 2.5 Risk of bias assessment

The quality assessment used the Newcastle–Ottawa Scale (NOS) to assess the quality of the included studies.^[11]^ The evaluation items consisted of 3 main items: subject selection, comparability, and outcome (cohort study) or exposure (case-control), and each item had evaluation entries, each of which was indicated by a ☆ when appropriate, with a maximum of 2☆ for comparability. Currently, the NOS scale has been more frequently used to evaluate case-control studies and cohort studies. The total score is 9 points, and NOS score ≥6 is divided into high-quality research.^[11]^ Two researchers (Li Yongkai and He Xiaojing) assessed the quality of all included studies and discussed discrepancies until consensus was reached.

## 3 Results

**Figure 1:**
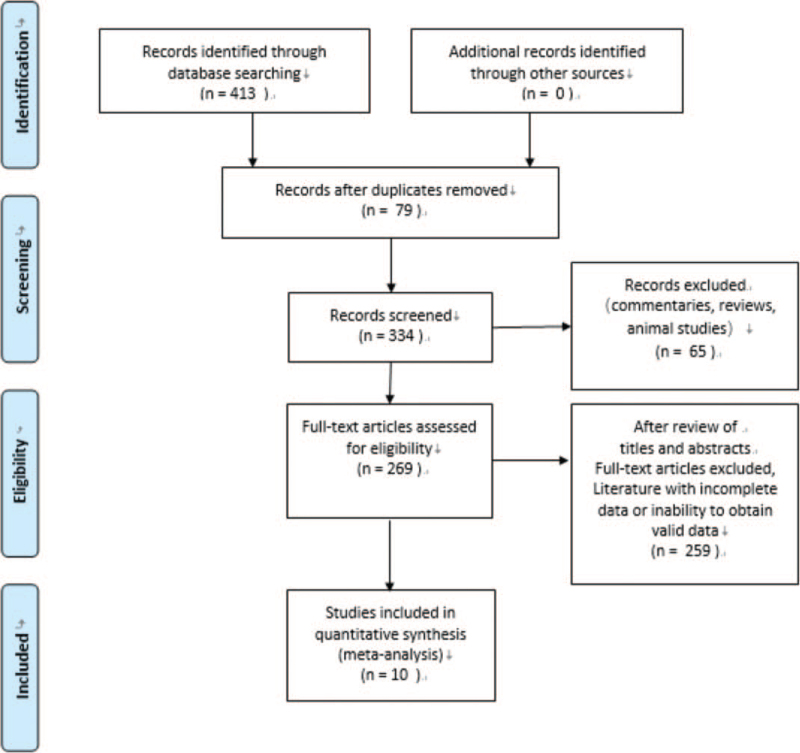
Process of study selection for the meta-analysis.

Our search initially identified 413 records. After removing duplicate studies, 334 studies remained. The exclusion of 65 studies from reviews, commentaries, and animal studies left 269 studies. A total of 259 studies were excluded after review of titles and abstracts due to full text of articles, incomplete data, or unavailability of valid data. Ten studies were finally included (Fig. 1), all 10 studies were published in 2020, all studies were conducted in China, and a total of 4248 patients with COVID-19 were included in the 10 publications, and the literature screening process and results are shown in (Table 1). The NOS scores of the included studies were 7 to 8, and all were of high quality, as detailed in (Table 1).

**Table 1:** Characteristics of individual studies included in the meta-analysis. Data were expressed as mean ± standard deviation and median (IQR).

First Author	Year	Research method	Patients	Severity	Patients	Gender (male/female)	Age	Serum amyloid A (mg/L)	Quality assessment
Dan Wang^[17]^	2020	Retrospective study	143	Mild/moderate	72	29/43	44 (32–60)	40.6 (13.6–141.0)	7
				Severe/critical	71	44/27	65 (53–69)	477.7 (209–996)	
Huan Li^[7]^	2020	Retrospective analysis	132	Mild/moderate	60	28/32	57.32 ± 11.52	123.57 ± 75.81	7
				Severe	56	37/19	66.55 ± 12.05	171.91 ± 56.89	
				Critically ill	16	10/6	64.06 ± 13.36	181.00 ± 40.66	
Jun Fu^[12]^	2020	Retrospective analysis	35	Mild	22	11/11	40.77 ± 9.06	89.78 ± 54.75	7
				Severe	13	2/11	60.08 ± 15.51	144.29 ± 57.33	
Lu Li^[13]^	2020	Observational study	72	Mild	22	12/10	22–61 (39.1 ± 12.2)	96.53 ± 31.00	8
				Moderate	38	20/18	24–71 (47.8 ± 14.4)	148.94 ± 54.58	
				Severe	12	7/5	28–71 (52.1 ± 14.2)	260.58 ± 42.67	
Qian Liu^[14]^	2020	Cross-sectional study	84	Nonsevere	59	31/28	49 (33–57)	14.70 (7.43–28.69)	8
				Severe	25	14/11	52 (45–67)	65.75 (14.30–117.80)	
Qianhui Zhang^[20]^	2020	Retrospective analysis	74	Nonsevere	47	18/29	61 (54–67)	10.84 (5.99–55.15)	8
				Severe	27	18/9	72 (58–81)	106.05 (52.05–167.62)	
S.-L. Liu^[15]^	2020	Retrospective analysis	225	Nonsevere	194	91/103	43.00 (33.00–57.00)	3.91 (1.00–18.79)	8
				Severe	31	17/14	64.00 (45.00–66.00)	48.57 (9.30–469.16)	
Shi Y^[16]^	2020	Observational study	164	Ordinary	150	66/84	3–82	25.78 (4.63∼156.15)	7
				Severe	8	4/4	51–81	200 (187.35∼250.33)	
				Critically ill	6	5/1	58–84	234.77 (174.28∼298.38)	
Xia Xintian^[18]^	2020	Retrospective analysis	63	Mild	32	15/17	62.25 ± 15.07	176.585 (37.833–300.000)	7
				Severe	31	18/13	64.55 ± 14.88	300.000 (194.830–300.000)	
Yalan Yu^[19]^	2020	Cohort study	3265	Mild	239	–	–	5.42 (4.39–7–87)	7
				Ordinary	1876			13.30 (6.09–113.80	
				Severe	862			24.74 (5.00–129.30	
				Critically ill	288			117.40 (72.76–197.10)	

IQR = interquartile range.

The 10 included studies in the literature were classified according to the severity of the novel coronavirus diagnosis and treatment guidelines, categorizing mild/moderate as nonsevere and severe/critical as severe,^[2]^ and the data were combined by meta-analysis using the method of combining the standard deviations of multiple subgroups. Severe and nonsevere were finally divided into 2 groups, and the combined data were meta-analyzed according to the SMD.^[10]^

The 10 studies in this study,^[^7^,^^12^^–^20^]^ after the heterogeneity test, I^2^ = 90% > 50% and *P* < .1 for the Q-test, suggest that there is strong heterogeneity among the literature selected for this study, and random effects can be selected for meta-analysis, and the reasons for heterogeneity can also be continued to be examined. Based on the data situation of this study, it is highly suspected that the source of heterogeneity is the elimination of this article Yu et al^[19]^ due to excessive sample resulting in large errors after converting the data to means and standard deviations, and the grouping situation of the severity of this article Li and Chen^[13]^ and the large differences in the means of each group make the heterogeneity among the outcome variables as well, and the elimination of this article can eliminate the heterogeneity, but The article is of good quality and analysis by random effects can be included in this literature. Nine studies were finally included.^[^7^,^^12^^–^18^,^^20^^]^ The study focused on a meta-analysis of the prognostic value of SAA on COVID-19, and only 2 of the 10 included papers (Qianhui Zhang 2020, Dan Wang 2020) showed that age and gender suggested statistical significance in terms of disease severity. In addition, the analysis of “Yalan Yu 2020” revealed a large heterogeneity, and the original data of this study did not include gender and age data, so this study was excluded. And other studies were analyzed in subgroups. However, the effects of gender and age on SAA severity in the other studies were not statistically analyzed, so it can be concluded that the 9 papers were grouped based on the same level of study on the severity of SAA and there was no effect of age and gender covariates on the overall effect size of SAA severity. Therefore, the effect of gender and age on the overall effect can be excluded. Further exploration of meta-analysis with random effects models is supported.

## 4 Discussion

### 4.1 Heterogeneity test

**Figure 2:**
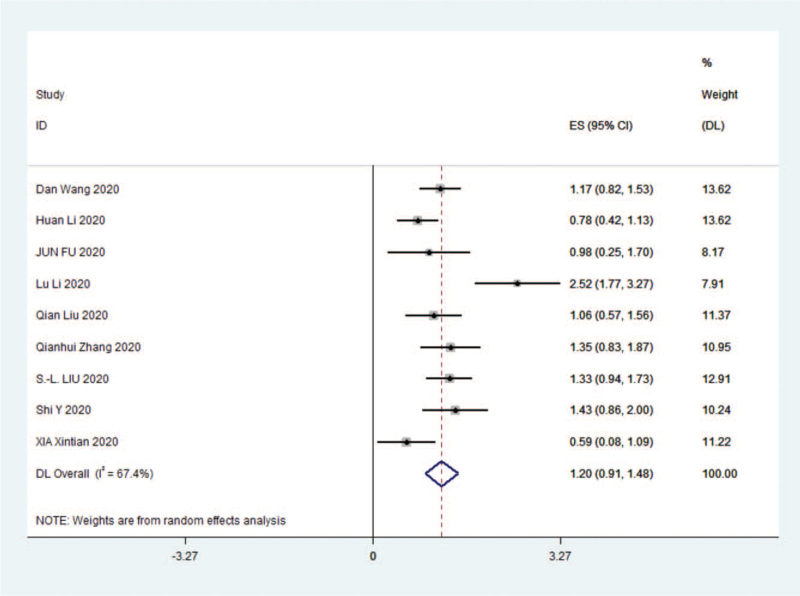
Forest plot comparing the SAA of the severe group with the nonsevere group in COVID-19. CI = confidence interval, COVID-19 = coronavirus disease 2019, ES = effect size.

For the 9 studies,^[^7^,^^12^^–^18^,^^20^^]^ data were analyzed by Stata 14.0 software and random effects were selected for meta-analysis, (SMD 1.20 [95% confidence interval {CI} 0.91–1.48]; I^2^ = 67.4%) and were statistically significant, z = 8.22 *P* < .01. The results were as follows (Fig. 2).

The results of the meta-analysis given by random effects showed that SAA levels were significantly higher in severe vs nonsevere (SMD 1.20 [95% CI 0.91–1.48]), which was statistically significant (*P* < .01).

**Figure 3:**
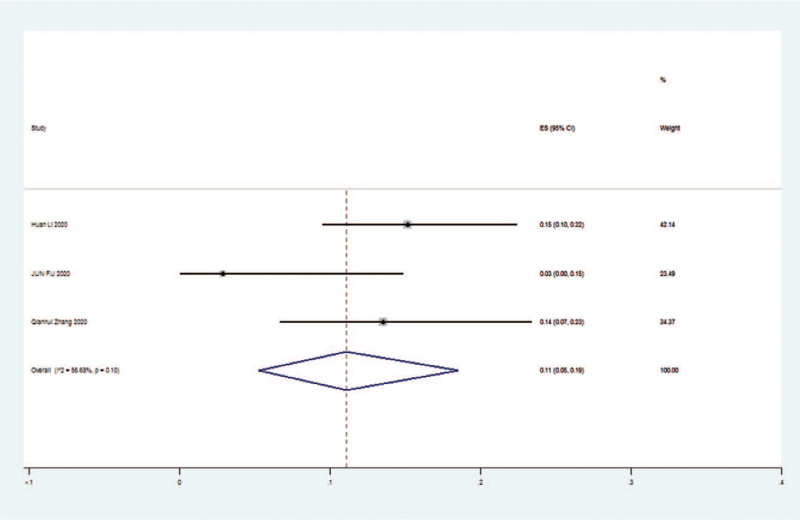
Risking of death in the severe and nonsevere groups of patients with COVID-19. CI = confidence interval, COVID-19 = coronavirus disease 2019, ES = effect size.

The 3 literatures studied,^[^7^,^^12^^,^^20^^]^ tested for heterogeneity, I^2^ = 56.66% > 50%, Q-test *P* = .1, indicating that there is significant heterogeneity between the literatures selected for this study, reaching moderate heterogeneity, so sensitivity analysis was continued to investigate the causes of heterogeneity. The 3 literatures studied^[^7^,^^12^^,^^20^^]^ (random effect size 0.11 [95% CI 0.05–0.19]; I^2^ = 56.68%) and were statistically significant, z = 5.46 *P* < .01, suggesting that the risk of death occurs at higher levels with increasing SAA values, with the risk of death in the severe group being 11% higher than in the nonsevere group. Details are shown in the following forest plot (Fig. 3).

### 4.2 Sensitivity analysis

**Figure 4:**
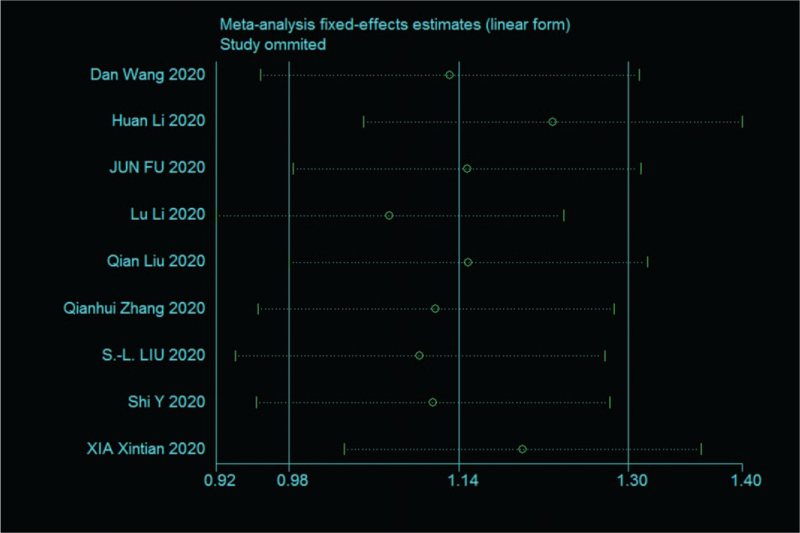
Sensitivity analysis of 9 literature severity groups and nonsevere groups in SAA.

Sensitivity analysis was performed on the 9 papers in this study,^[^7^,^^12^^–^18^,^^20^^]^ and none of them caused much interference to the results of this meta-analysis, implying that this study has good stability. The details are shown in the following (Fig. 4).

**Figure 5:**
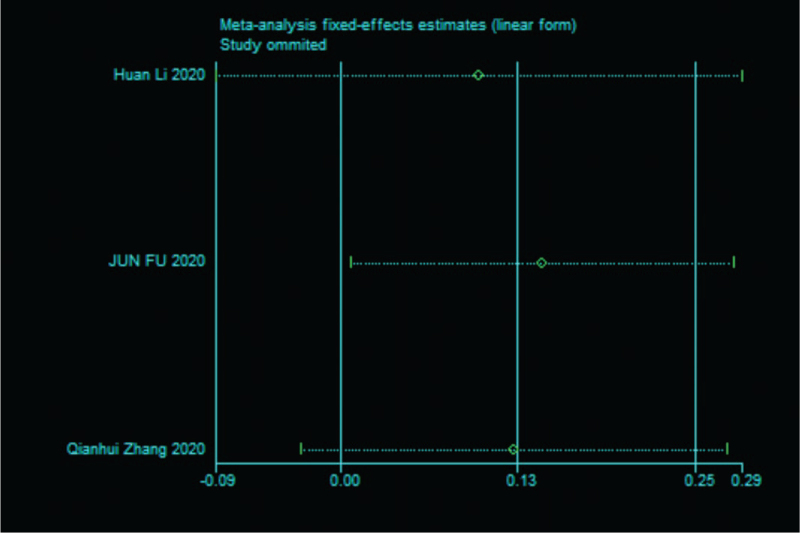
Sensitivity analysis of death risk in 3 studies.

Sensitivity analysis was performed on the 3 papers in this study,^[^7^,^^12^^,^^20^^]^ and none of them caused significant interference to the results of this meta-analysis, implying that this study has good stability. The details are shown in the following (Fig. 5).

### 4.3 Bias test

**Figure 6:**
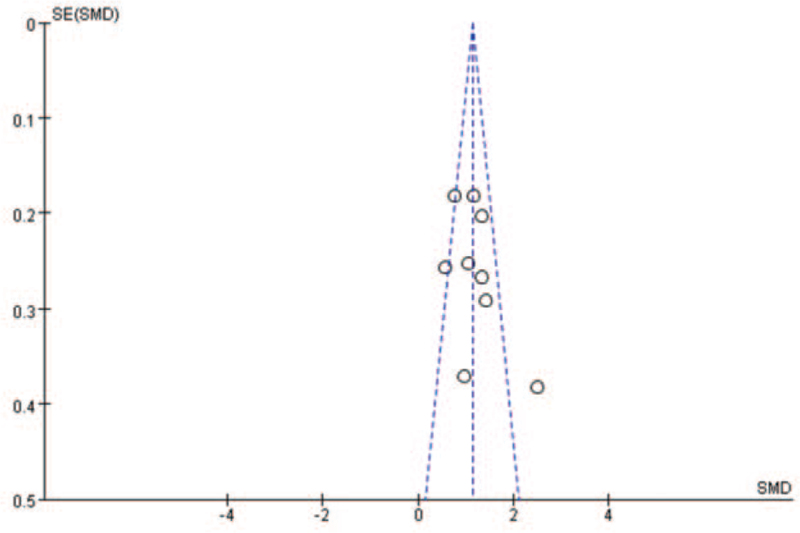
Nine papers performed funnel plot bias tests for SAA in the COVID-19 severe and nonsevere groups. COVID-19 = coronavirus disease 2019, SMD = standardized mean difference.

The data were analyzed by RevMan 5.4 software and funnel diagram were drawn with the following results (Fig. 6).

From the above figure, it can be clearly seen that the funnel plot of this study is basically symmetrical,^[^7^,^^12^^–^18^,^^20^^]^ while the bias test yielded: *P* > .01, metabias: *P* = .466 > .05 therefore it can be judged that there is no publication bias in the literature of this study.

**Figure 7:**
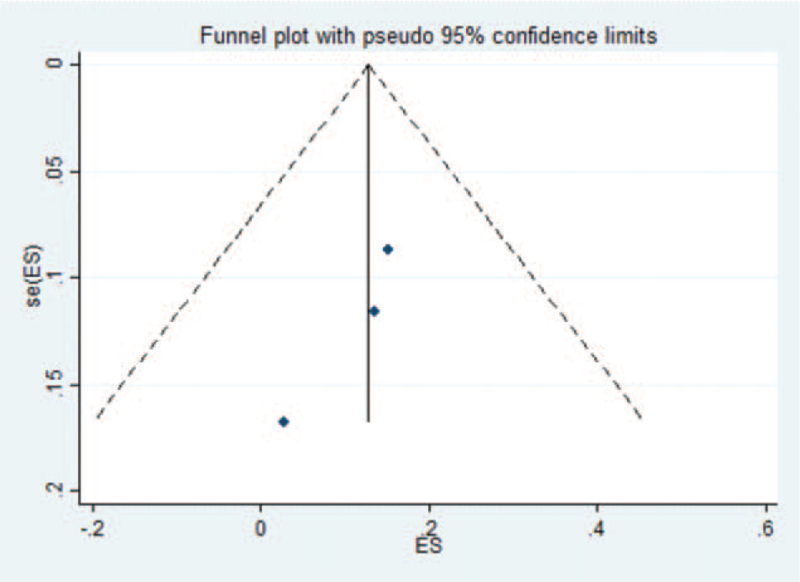
Three papers were tested for funnel plot bias in the risk of death. ES = effect size.

The data of 3 studies were analyzed by Stata software.^[^7^,^^12^^,^^20^^]^ The presence of publication bias in the current study was examined by plotting a funnel plot, which was symmetrical implying no publication bias. The funnel plot for the current study was as follows (Fig. 7).

The symmetry test on the above figure yielded *P* = .296 > .05, implying that the funnel plot is symmetrical and therefore it can be judged that there is no publication bias in the literature of this study.^[^7^,^^12^^,^^20^^]^

### 4.4 Limitations

Although this study was designed and reported according to standardized meta-analysis methods^[8]^ and used a detailed search strategy, there are important limitations to the study, many of which are inherent to the methodological quality of the included base studies. All base studies eligible for inclusion were conducted in China. Most of the included studies were retrospective and subject to confounding bias. Fewer studies of SAA in COVID-19 were reported, and the test efficacy may be insufficient.

## 5 Conclusions

In this meta-analysis, a total of 9 papers were included,^[^7^,^^12^^–^18^,^^20^^]^ and the results of the meta-analysis showed that the SAA of patients in the severe group were all higher than those in the nonsevere type group, and the analysis of the reasons may be: most of the chronic diseases have common features with infectious diseases, such as higher age and inflammatory response status, weakened immune function, etc. Through this study, it was found that as the risk of death occurred with higher SAA values, the risk of death in the severe group increased by 11% compared to the risk of death in the nonsevere group. SAA belongs to the apolipoprotein family, which is an acute-phase reactive protein, mainly from the It plays an important role in the inflammatory response and lipid metabolism. After infection, SAA can increase rapidly by about 1000-fold in the acute phase and then decrease rapidly to normal levels after the pathogen is cleared, which is a sensitive indicator of infection and inflammation recovery and that elevated SAA levels were significantly associated with adverse clinical outcomes. This suggests that COVID-19 is the result of a novel respiratory and systemic disease caused by SARS-CoV-2. Therefore, SAA can be considered as a biomarker for predicting the severity and prognosis of COVID-19. SAA can be used for early warning of poor prognosis of COVID-19 and for monitoring the recovery process, which has important clinical value.

## Author contributions

**Conceptualization:** Yongkai Li, Jianzhong Yang

**Data curation:** Yongkai Li, Xiaojing He

**Formal analysis:** Yongkai Li, Jianzhong Yang, Xiaojing He

**Investigation:** Zhuanyun Li, Dandan Li

**Methodology:** Yongkai Li, Xiaojing He

**Project administration:** Yongkai Li, Jianzhong Yang, Xiaojing He

**Resources:** Yongkai Li.

**Software:** Yongkai Li, Xiaojing He

**Supervision:** Yongkai Li.

**Validation:** Yongkai Li.

**Visualization:** Yongkai Li.

**Writing – original draft:** Yongkai Li, Xiaojing He

**Writing – review & editing:** Yongkai Li, Jianzhong Yang, Xiaojing He
